# Orosomucoid 2 inhibits tumor metastasis and is upregulated by CCAAT/enhancer binding protein β in hepatocellular carcinomas

**DOI:** 10.18632/oncotarget.3867

**Published:** 2015-04-19

**Authors:** Tao Fang, Meiling Cui, Ji Sun, Chao Ge, Fangyu Zhao, Lin Zhang, Hua Tian, Lixing Zhang, Taoyang Chen, Guoping Jiang, Haiyang Xie, Ying Cui, Ming Yao, Hong Li, Jinjun Li

**Affiliations:** ^1^ State Key Laboratory of Oncogenes and Related Genes, Shanghai Cancer Institute, Renji Hospital, Shanghai Jiaotong University School of Medicine, Shanghai, China; ^2^ Shanghai Medical Colloge, Fudan University, Shanghai, China; ^3^ Qi Dong Liver Cancer Institute, Qi Dong, Jiangsu Province, China; ^4^ Department of General Surgery, The First Affiliated Hospital, School of Medicine, Zhejiang University, Hangzhou, China; ^5^ Cancer Institute of Guangxi, Nanning, China

**Keywords:** hepatocellular carcinoma, ORM2, C/EBPβ, metastasis

## Abstract

Cancer metastasis is a complex process, and the incidence of metastasis is influenced by many biological factors. Orosomucoid 2 (ORM2) is an important glycoprotein that is mainly biosynthesized and secreted by hepatocytes. As an acute-phase protein, ORM2 likely plays important roles in anti-inflammation, immunomodulation and drug delivery. However, little is known regarding the function of ORM2 in hepatocellular carcinoma (HCC). In this study, we determined that ORM2 expression in HCC tissues was negatively associated with intrahepatic metastasis and histological grade. Moreover, the ectopic overexpression of ORM2 decreased HCC cell migration and invasion *in vitro* and intrahepatic metastasis *in vivo*, whereas silencing ORM2 expression resulted in increased tumor cell migration and invasion *in vitro*. The CCAAT/enhancer binding protein β (C/EBPβ) upregulated ORM2 expression, while only the LAP1/2 (C/EBPβ isoforms) possessed transcription-promoting activity on the ORM2 promoter. Subsequently, we found that LAP1 repressed HCC cell migration and invasion via the induction of ORM2 expression. Consistently, the protein expression of C/EBPβ was negatively associated with histological grade and positively correlated with ORM2 protein expression in HCC tissues. Collectively, our findings indicate that ORM2 is a functional downstream target of C/EBPβ and functions as a tumor suppressor in HCC.

## INTRODUCTION

Hepatocellular carcinoma (HCC) is one of the most common malignant cancers worldwide. Most patients with HCC have a poor prognosis, largely due to a high rate of postsurgical recurrence and metastasis [1]. Cancer metastasis is a complex process, and the incidence of metastasis is influenced by many biological and environmental factors. Hence, there is an urgent need to identify metastatic factors and elucidate the underlying molecular mechanisms that are involved in HCC metastasis [2].

Orosomucoid (ORM), also known as alpha 1 acid glycoprotein, is an important glycoprotein with a molecular weight of 41-43 kDa. The ORM family includes two main members: ORM1 and ORM2 [3]. ORM2 is a type of acute-phase protein and is mainly biosynthesized and secreted by hepatocytes. ORM2 is generally considered an anti-inflammatory and immunomodulatory factor due to its anti-neutrophil and anti-complement activity [4]. ORM2 also depresses cytokine secretion by an unknown mechanism to protect human health [5]. Additionally, ORM serves as a bimodal regulator of angiogenesis and enhances capillary permeability in guinea pig skin [6, 7]. Other studies have indicated that ORM inhibits endothelial cell capillary-like tube formation in a manner that may be explained by diminished cell adhesion to the underlying matrix and/or reversible decreases in cell migration [8], and ORM can decrease microvascular permeability as well as tumor cell adhesion [9]. This emerging evidence suggests that ORM2 may play important roles in tumor metastasis and progression. ORM2 expression positively correlates with the progression of colorectal cancer [10, 11] and lung cancer [12, 13]. Moreover, ORM2 is believed to be a potential biomarker for cholangiocarcinoma in combination with kinesin 18A [14]. However, the function of ORM2 in HCC and the relationship between its expression and clinicopathologic significance remain unclear.

Many external stimuli can upregulate ORM2 expression, including IL-6 and glucocorticoids [6]. Moreover, a binding motif for the CCAAT/enhancer binding protein β (C/EBPβ) has been identified in the promoter region of human *ORM2* [15]. C/EBPβ is an important regulator that has been implicated in many biological activities, including inflammatory responses, adiposeness and cancer progression. C/EBPβ exists as three isoforms (LAP1, LAP2 and LIP) that are translated by in-frame alternative translation initiation [16]. LAP1 and LAP2 are transcriptional activators, while LIP functions as a repressor through its antagonism for LAP [17]. LIP has been reported to be deregulated in breast cancer and to promote breast cancer invasion [18]. The loss of C/EBPβ regulation in breast cancer promotes malignant progression by shifting the TGF-β response from growth inhibition to epithelial to mesenchymal transition (EMT), invasion and metastasis [19]. Additionally, C/EBPβ expression is upregulated by Epidermal Growth Factor Receptor (EGFR) in oral carcinomas and esophageal squamous cell carcinomas [20, 21] due to endoplasmic reticulum (ER) stress in hepatoma cells [22]. However, the role of C/EBPβ in HCC tumorigenesis and how the three isoforms of C/EBPβ regulate ORM2 in HCC remain poorly understood.

In the present study, we found that ORM2 was significantly downregulated in HCC tissues and inhibited HCC cell metastasis. Additionally, we found that the LAP1/2 isoforms of C/EBPβ could upregulate ORM2 expression by directly binding to the *ORM2* promoter, thereby repressing HCC cell migration and invasion through the induction of ORM2 expression.

## RESULTS

### ORM2 is frequently downregulated in HCC tissues and is negatively associated with tumor progression and intrahepatic metastasis

**Figure 1:**
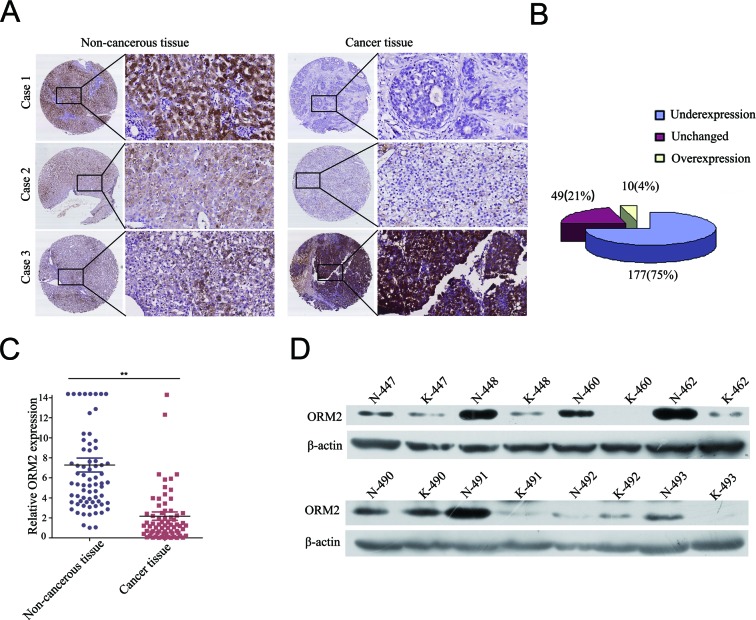
ORM2 was downregulated in HCC tissues and negatively associated with tumor progression and metastasis **A.** IHC analysis of ORM2 expression in HCC tissues compared with adjacent non-cancerous tissues (original magnification 40× and 200×). **B.** Statistical analysis of ORM2 expression in HCC samples between HCC tissues and adjacent non-cancerous tissues. **C.** qRT-PCR was performed to detect ORM2 expression in 70 pairs of HCC tissues. **D.** Western blotting of ORM2 protein levels in 30 pairs of HCC tissues (K) and the corresponding adjacent non-cancerous liver tissues (N). Representative images are shown. β-actin was used as a loading control. **, *p* < 0.01.

To investigate the clinicopathological role of ORM2 in HCC progression, we determined the expression levels of the ORM2 protein in 236 pairs of HCC tissues and matched non-tumorous liver tissues using immunohistochemistry staining (IHC). Out of the 236 cases, 177 cases (75%) had lower ORM2 protein expression in HCC tissues compared with their corresponding non-tumorous liver tissues, 49 (20.76%) cases had similar expression, and only 10 (4.24%) HCC patients had higher expression in cancer tissues (Figure 1A and 1B). Therefore, the results showed that ORM2 was frequently downregulated in primary HCC tissues compared with the adjacent non-cancerous liver tissues. qRT-PCR and Western blot assays further verified that ORM2 expression was downregulated in HCC tissues (Figure 1C and 1D).

Based on the IHC results, the expression intensity of the ORM2 protein was scored as 0 or 1 for weak or strong immunostaining, respectively. The analysis showed that ORM2 expression was negatively associated with the histological grade of HCC (*p* = 0.013) and the presence of intrahepatic metastasis (*p* = 0.024). However, there was no correlation between ORM2 expression and other clinicopathological factors, such as age, gender, tumor size, the presence of cirrhosis, serum alpha-fetoprotein (AFP) and hepatitis B surface antigen (HBsAg) levels (Table 1). Taken together, our results suggest that the loss of ORM2 expression might contribute to HCC progression and metastasis.

**Table 1:** Relationship between ORM2 protein expression and Clinicopathological features in HCC tissues

		ORM2 Immunostaining		
Clinicopathological Features	Number of cases	Score 0 N (%)	Score 1 N (%)*P*	*p* Value
Age (years)				
<60	159	77(70.64)	82(65.08)	0.363
≥60	76	32(29.36)	44(34.92)	
Gender				
Male	190	87(79.09)	103(81.75)	0.607
Female	46	23(20.91)	23(18.25)	
Tumor size				
≤5cm	113	53(50.00)	60(48.78)	0.854
>5cm	116	53(50.00)	63(51.22)	
AFP (ng/ml)				
≤20	79	34(31.48)	45(36.29)	0.441
>20	153	74(68.52)	79(63.71)	
HBV infection				
Negative	42	16(15.09)	26(21.14)	0.239
Positive	187	90(84.91)	97(78.86)	
Cirrhosis				
Absent	38	16(17.02)	22(17.46)	0.543
Present	198	94(82.92)	104(82.54)	
Edmondson's grade				
I, II	119	46(41.82)	73(57.94)	0.013*
III, IV	117	64(58.18)	53(42.06)	
Intrahepatic metastasis				
Absent	161	67(60.91)	94(74.60)	0.024*
Present	75	43(39.09)	32(25.40)	

AFP, alpha-fetoprotein. N, Number of cases. *P* value represents the probability from a Chi-square test for different immunohistochemical scores of ORM2 in HCC tissues.

* *p*<0.05.

### ORM2 significantly inhibits HCC cell migration and invasion *in vitro* and metastasis *in vivo*

Next, we measured ORM2 mRNA expression in HCC cell lines (Supplementary Figure S1). ORM2 expression was upregulated only in Huh7 and PLC/PRF/5 cells, whereas in other cells, ORM2 expression was barely detectable. These results were consistent with the pattern of ORM2 protein expression in HCC tissues, where ORM2 protein expression was barely detectable by Western blot or IHC.

**Figure 2:**
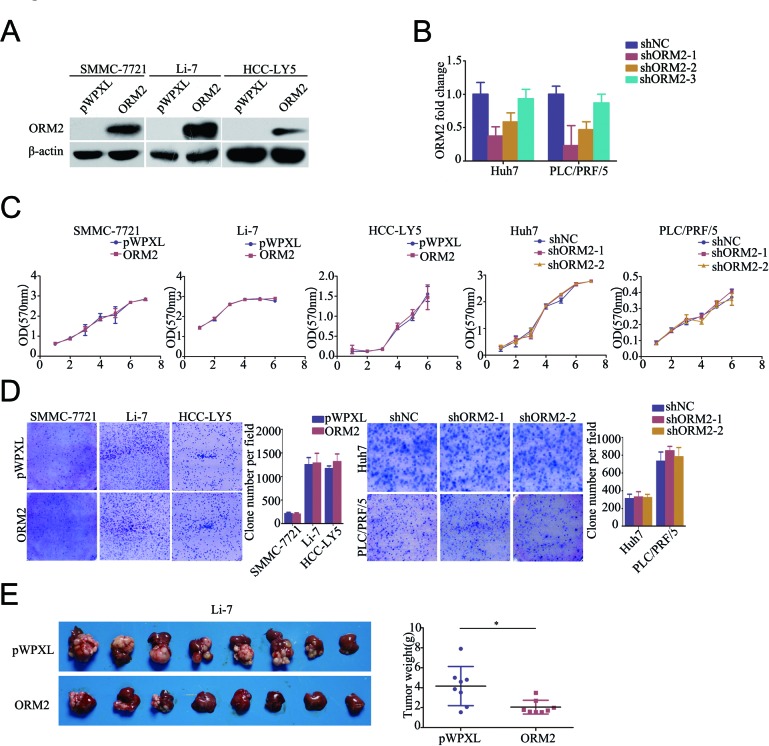
ORM2 inhibited HCC cell growth *in vivo* **A.** Western blotting of ORM2 protein levels in SMMC-7721, Li-7 and HCC-LY5 cells stably transfected with ORM2 or the control. **B.** qRT-PCR of ORM2 mRNA levels in Huh7 and PLC/FRP/5 cells transfected with shORM2 or the negative control (shNC). **C.** A MTT assay was performed in the SMMC-7721, Li-7, HCC-LY5, Huh7, and PLC/PRF/5 cell lines stably transfected with ORM2, sh-ORM2, or the control. **D.** Clone formation was performed in the SMMC-7721, Li-7, HCC-LY5, Huh7, and PLC/PRF/5 cell lines stably transfected with ORM2, shORM2, or the control. **E.** Li-7 cells stably expressing ORM2 were injected orthotopically into nude mice; empty vectors were used as a control. The tumors were removed from the nude mice after 4 weeks. Representative images are shown, along with the weight of the livers with tumors. *, *p* < 0.05.

To better understand the function of ORM2 in HCC, we constructed a lentivirus vector containing the complete ORF of ORM2 and established the SMMC-7721-ORM2, Li-7-ORM2 and HCC-LY5-ORM2 HCC cell lines; cells infected with an empty vector were used as controls (Figure 2A). We also designed three shRNAs using lentivirus vectors to specifically knock down endogenous ORM2 in the Huh7 and PLC/FRP/5 cell lines and selected two effective shRNAs (shORM2-1 and shORM2-2) for further assays (Figure 2B). MTT and colony formation assays indicated that ORM2 had no notable effect on HCC cell growth *in vitro* (Figure 2C and 2D), while ORM2 overexpression obviously repressed orthotopic tumor growth *in vivo* (Figure 2E).

**Figure 3:**
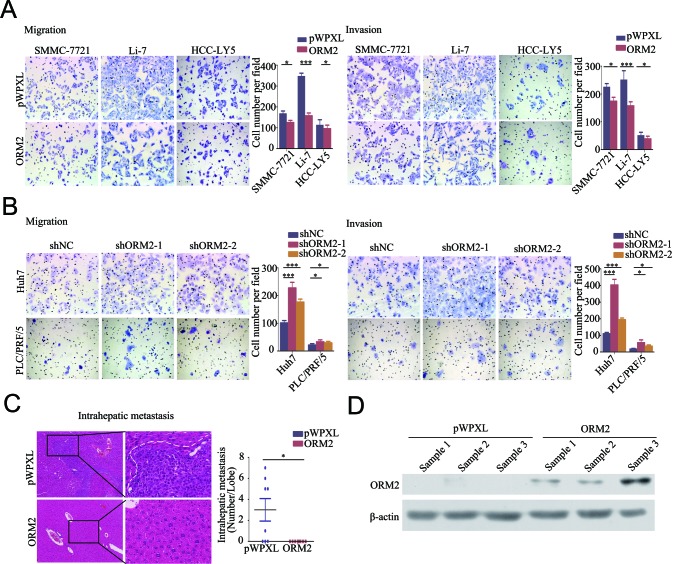
ORM2 inhibited HCC cell migration and invasion *in vitro* and *in vivo* **A.** The *in vitro* migration and invasion ability of SMMC-7721 and Li-7 cells transfected with ORM2 were assessed using transwell assays; cells containing the empty vector were used as a control. **B.** The *in vitro* migration and invasion ability of Huh7 and PLC/PRF/5 cells transfected with shORM2 were assessed using transwell assays; shNC was used as a control. **C.** Representative images of intrahepatic metastatic nodules formed by Li-7 cells transfected with ORM2 or the control are shown; the image consists of sections stained with hematoxylin and eosin (HE) (original magnification: left images, 40×; right images, 200×). The numbers of intrahepatic metastatic nodules are shown in the right images. **D.** Western blotting of ORM2 protein levels in xenografts. *, *p* < 0.05, **, *p* < 0.01, ***, *p* < 0.001.

As mentioned above, the clinical data showed that ORM2 is involved in HCC intrahepatic metastasis. Therefore, we performed *in vitro* transwell migration and invasion assays to assess the effect of ORM2 on the spreading metastatic potential of HCC cells. The results showed that stable overexpression of ORM2 significantly suppressed the *in vitro* migration and invasion of SMMC-7721, Li-7 and HCC-LY5 cells compared with the controls (Figure 3A), while the knockdown of endogenous ORM2 markedly increased the migration and invasion of Huh7 and PLC/FRP/5 cells (Figure 3B). These results suggest that ORM2 significantly inhibits HCC cell migration and invasion *in vitro*.

To further clarify the role of ORM2 in HCC metastasis *in vivo*, Li-7-ORM2 and the control cells (Li-7-pWPXL) were orthotopically inoculated into the left hepatic lobes of mice using a microsyringe. Histological examination of liver tissue sections indicated that tumor-bearing mice with ORM2 overexpression had fewer intrahepatic metastatic nodules than control mice (*p* < 0.05, Figure 3C). Therefore, these results indicate that ORM2 suppresses HCC intrahepatic metastasis *in vivo*.

### LAP1/2 directly binds to the ORM2 promoter and promotes ORM2 expression in HCC cells

**Figure 4:**
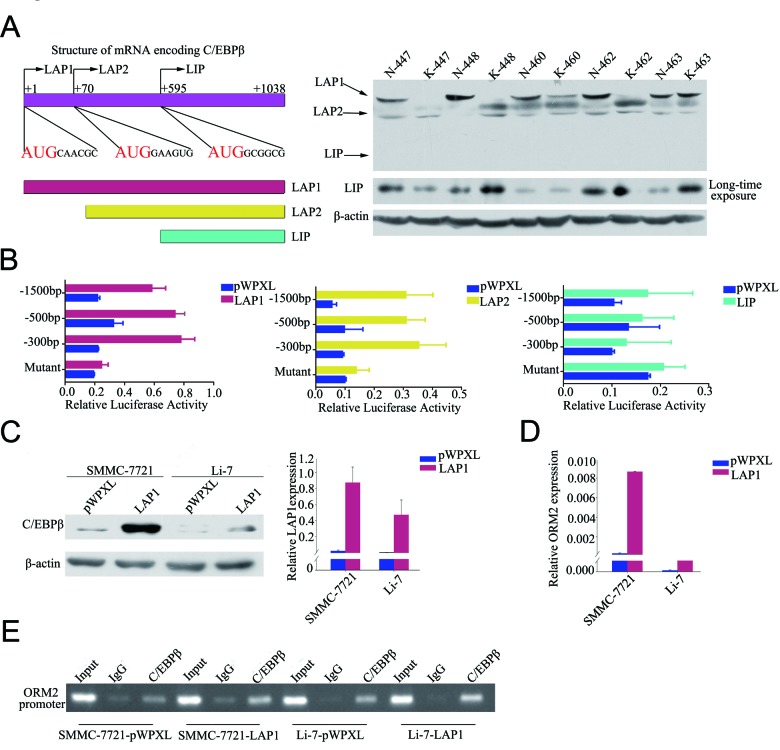
LAP1/2 directly binds to the ORM2 promoter and promotes ORM2 expression in HCC cells **A.** The left lane showed schematic representation of the three isoforms of C/EBPβ. The three isoforms had the same mRNA and are translated by in-frame alternative translation initiation at different AUG start codons. The right lane showed C/EBPβ expression at the protein level was determined by Western blotting in HCC tissues. **B.** The promoter activity of ORM2 was determined after transfection with LAP1, LAP2 and LIP. **C.** Western blotting of C/EBPβ protein levels and qRT-PCR assay of C/EBPβ mRNA levels in SMMC-7721 and Li-7 cells stably transfected with C/EBPβ or the control. **D.** qRT-PCR assay of ORM2 mRNA levels of SMMC-7721 and Li-7 cells stably transfected with C/EBPβ or the control. **E.** Binding of C/EBPβ to the ORM2 promoter was assayed by ChIP performed using an antibody against C/EBPβ in SMMC-7721 and Li-7 cells. A negative control with IgG was included for comparative analysis. *, *p* < 0.05, **, *p* < 0.01, ***, *p* < 0.001.

To explore the regulation of ORM2 in HCC cells, the promoter region of ORM2 was analyzed using the TFSEARCH program. A C/EBPβ binding site located at −48/−32bp of the ORM2 promoter region was found, consistent with a previous report [15]. As mentioned above, C/EBPβ has three major isoforms that possess different functions. Therefore, we measured the expression of the three isoforms in 30 pairs of HCC specimens by Western blotting. The results showed that the LAP1 protein was significantly downregulated in HCC tissues compared with matched non-cancerous liver tissues; no changes were observed in LAP2 protein levels, and LIP protein expression was low or undetectable (Figure 4A). Among these isoforms, LAP1 and LAP2 have the same transcription factor binding site and are generally considered to activate translation, while LIP is believed to inhibit translation by antagonizing LAP [23]. To verify the function of these isoforms in the regulation of ORM2, lentivirus vectors containing the complete ORF of LAP1, LAP2 or LIP were constructed, and the activity of the ORM2 promoter was examined using luciferase reporter assays in cells overexpressing the three isoforms. The results showed that ORM2 promoter activity was enhanced when LAP1 or LAP2 were stably overexpressed, while LIP overexpression had no obvious effect on the ORM2 promoter activity in HCC cells (Figure 4B). ORM2 promoter activity was not enhanced by co-transfection of LAP1 or LAP2 with a mutant ORM2 promoter containing a deletion of the C/EBPβ binding site (Figure 4B). Thus, ORM2 expression was obviously increased when LAP1 or LAP2 was overexpressed in the SMMC-7721 and Li-7 HCC cell lines (Figure 4C, 4D and Supplementary Figure S2). ChIP assays further confirmed that C/EBPβ directly bound to the *ORM2* promoter region in HCC cells (Figure 4E). Collectively, these results indicate that LAP1/2 increased ORM2 expression via direct binding to the ORM2 promoter in HCC cells.

### C/EBPβ is often downregulated in HCC tissues and LAP1 represses HCC cell migration and invasion *in vitro*

**Figure 5:**
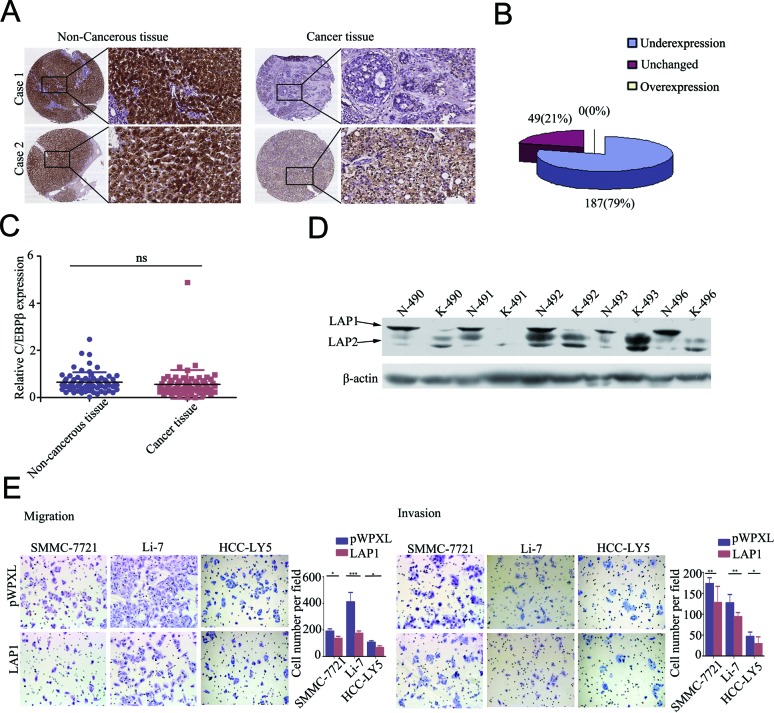
LAP1 represses HCC cell migration and invasion ability *in vitro* **A.** IHC analysis of C/EBPβ expression in HCC tissues compared with paired non-cancerous tissues (original magnification: left pictures, 40×; right pictures, 200×). **B.** Statistical analysis of C/EBPβ expression in HCC tissues and adjacent non-cancerous tissues. **C.** qRT-PCR was performed to detect the expression of C/EBPβ in 70 pairs of HCC tissues. **D.** Western blotting of C/EBPβ protein levels in HCC tissues (K) and the corresponding adjacent non-cancerous liver tissues (N). **E.** The migration and invasion ability of SMMC-7721, Li-7 and HCC-LY5 cells transfected with C/EBPβ were assessed by transwell assays; cells transfected with the empty vector were used as a control. *, *p* < 0.05, **, *p* < 0.01, ***, *p* < 0.001.

We analyzed C/EBPβ protein expression in 236 pairs of human primary HCC tissues and matched non-tumorous liver tissues in microtissue arrays using IHC. The results demonstrated that C/EBPβ expression was lower in HCC tissues compared with non-tumorous liver tissues, with 79.24% (187/236) C/EBPβ protein staining in HCC tissues compared with 20.76% (49/236) of non-tumorous liver tissues displaying staining to a similar extent (Figure 5A and 5B). Therefore, the results showed that C/EBPβ was frequently downregulated in primary HCC tissues compared with adjacent non-tumorous liver tissues. To verify the result, we performed qRT-PCR and Western blotting to analyze C/EBPβ mRNA levels and protein expression levels. The expression of the LAP1 protein was downregulated in HCC tissues compared with adjacent non-cancerous liver tissues (Figure 5D), but no significant difference in C/EBPβ mRNA expression was observed (Figure 5C). Bundy *et al* speculated that the production of the three isoforms from the same C/EBPβ mRNA may be one mechanism to explain the differences in mRNA expression and protein expression [24].

Based on the IHC results, the expression intensity of the C/EBPβ protein was scored as 0 or 1 for weak or strong immunostaining, respectively. The results showed that C/EBPβ expression was negatively associated with gender (*p* = 0.02) and HCC histological grade (*p* = 0.002). However, there was no correlation between C/EBPβ expression and other clinicopathological factors, such as age, tumor size, intrahepatic metastasis (*p* = 0.074), the presence of cirrhosis, and serum AFP and HBsAg levels (Table 2).

**Table 2:** Relationship between C/EBPβ protein expression and Clinicopathological features in HCC tissues

		C/EBPβ immunostaining		
Clinicopathological Features	Number of cases	Score 0 N (%)	Score 1 N (%)	*p* Value
Age (years)				
<60	159	100(69.44)	59(64.84)	0.462
≥60	76	44(30.56)	32(35.16)	
Gender				
Male	190	109(75.69)	81(88.04)	0.020*
Female	46	35(24.31)	11(11.96)	
Tumor size				
≤5cm	113	72(51.80)	41(45.55)	0.356
>5cm	116	67(48.20)	49(54.45)	
AFP (ng/ml)				
≤20	79	42(29.79)	37(40.66)	0.088
>20	153	99(70.21)	54(59.34)	
HBV infection				
Negative	42	30(21.43)	12(13.48)	0.130
Positive	187	110(78.57)	77(86.52)	
Cirrhosis				
Absent	38	19(13.20)	19(20.65)	0.128
Present	198	125(86.80)	73(79.35)	
Edmondson's grade				
I, II	119	61(42.36)	58(63.04)	0.002*
III, IV	117	83(57.64)	34(36.96)	
Intrahepatic metastasis				
Absent	161	92(63.89)	69(75.00)	0.074
Present	75	52(36.11)	23(25.00)	

AFP, alpha-fetoprotein. N, Number of cases. *P* value represents the probability from a Chi-square test for different immunohistochemical scores of C/EBPβ in HCC tissues.

* *p*<0.05.

As mentioned above, LAP1 and LAP2 play redundant roles in the regulation of ORM2, while LIP has no effect on ORM2. These results suggest that among the three isoforms, LAP1/2 functions as a major regulator of ORM2 in HCC cells. Therefore, we further investigated the function of LAP1/2 in HCC cells. The results of *in vitro* transwell migration and invasion assays showed that LAP1 overexpression inhibited the migration and invasion potential of SMMC-7721 and Li-7 HCC cells (Figure 5E). Additionally, the stable overexpression of LAP2 inhibited the *in vitro* migration and invasion potential of SMMC-7721 and Li-7 HCC cells (Supplementary Figure S3). Therefore, our results showed that LAP1 inhibits the *in vitro* migration and invasion of HCC cells.

### LAP1 represses *in vitro* HCC cell migration and invasion by inducing ORM2 expression, and C/EBPβ expression positively correlates with ORM2 expression in HCC tissues

**Figure 6:**
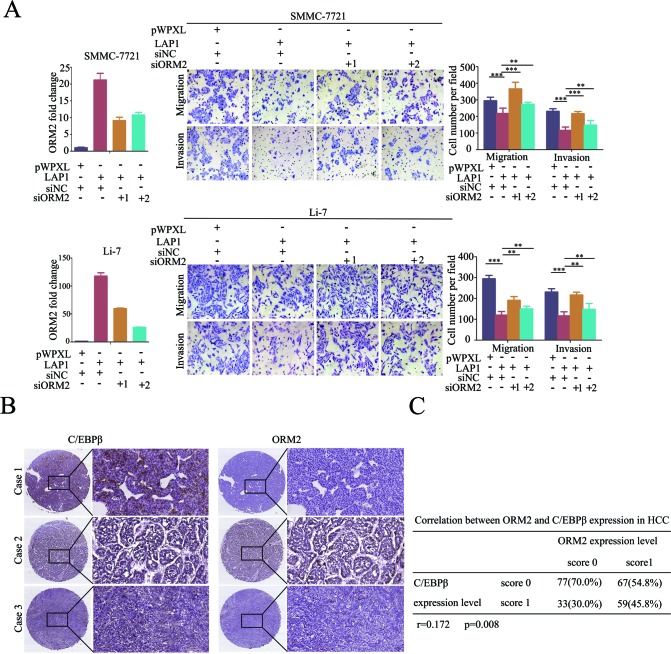
LAP1 represses HCC cell migration and invasion by inducing ORM2 expression **A.** SMMC-7721 and Li-7 cells overexpressing LAP1 were transiently transfected with siRNA targeted to ORM2 as indicated. QRT-PCR of ORM2 is shown in the left panel. The migration and invasion ability of the SMMC-7721 and Li-7 cells were assessed by transwell assays; cells transfected with the empty vector were used as a control. **B.** Representative immunostaining of C/EBPβ and ORM2 in HCC tissues (original magnification: left images, 40×; right images, 200×). **C.** The correlation between C/EBPβ and ORM2 protein expression in 236 HCC tissues was analyzed. *r* = 0.172, *p* = 0.008. **, *p* < 0.01, ***, *p* < 0.001.

To determine whether ORM2 is a functional downstream target of LAP1 in HCC, ORM2 expression was knocked down in SMMC-7721 and Li-7 HCC cells overexpressing LAP1. *In vitro* transwell migration and invasion assays showed that the stable overexpression of LAP1 inhibited the *in vitro* migration and invasion of SMMC-7721 and Li-7 HCC cells, whereas the knockdown of ORM2 antagonized the inhibition of the *in vitro* migration and invasion by LAP1 compared with the controls (Figure 6A). The results indicate that ORM2 is a functional downstream target of LAP1 in HCC.

Next, we analyzed the relationship between C/EBPβ and ORM2 expression in HCC. The results showed that there was a positive correlation between the protein expression levels of C/EBPβ and ORM2 in HCC tissues (*r* = 0.172, *p* = 0.008; Figure 6B and 6C). Further analysis demonstrated that out of the 187 HCC tissues, in which C/EBPβ expression was downregulated compared with non-tumorous liver tissues, 75.40% (141/187) of cases also had lower ORM2 protein expression compared with non-tumorous liver tissues, 20.32% (38/187) of cases had similar expression, and only 4.28% (8/187) had higher expression in HCC tissues. Taken together, our findings suggest that ORM2 is a functional downstream target of LAP1 and C/EBPβ expression positively correlates with ORM2 expression in HCC tissues.

## DISCUSSION

ORM2, also known as an acute-phase protein, possesses anti-inflammatory and immunomodulatory properties [11, 25-27]. ORM2 inhibits neutrophil migration, and inhibition of ORM2 expression results in the high expression of CD11b in the neutrophils of diabetic mice [4, 28]. It is well known that inflammation plays decisive roles at different stages of tumor development, including initiation, promotion, malignant conversion, invasion, and metastasis [29]. Therefore, these experimental findings led us to hypothesize that ORM2 contributes to HCC progression.

In the present study, we demonstrated that ORM2 was frequently downregulated in primary HCC tissues and negatively correlated with intrahepatic metastasis and histological grade. Further studies showed that ORM2 inhibited cell migration, invasion, and metastasis *in vitro* and *in vivo* and repressed the growth of orthotopically implanted tumors *in vivo*. ORM is required for the maintenance of normal permselectivity of the capillary walls [30], decreased microvascular permeability and reduced adhesion of the MDA-MB-231 mammary tumor cells to vessel walls [9]. Tumor cell adhesion to the microvascular wall is one of the critical steps in tumor metastasis [31]. Our data showed that stable overexpression of ORM2 inhibited HCC cell growth *in vivo* but not *in vitro*. ORM2 inhibits endothelial cell capillary-like tube formation [8] and possesses anti-inflammatory properties, which might help us to understand the differences between the *in vivo* and *in vitro* functions of the gene. The results of clinical pathological index analysis showed that serum ORM2 levels positively correlated with cancer progression [12] [32] [33]. Thus, more details are needed to elucidate the mechanism of how ORM2 affects tumor growth and metastasis in HCC and its different functions in different tumors.

Based on previous reports [34] and our current data, C/EBPβ is an important transcription factor for the regulation of ORM2 expression. Among its three isoforms (LAP1, LAP2, and LIP) [35], LAP1 and LAP2 are transcriptional activators, while LIP functions as a repressor due to its antagonism for LAP [17]. Because of the existence of three C/EBPβ isoforms, the function of C/EBPβ in cancer progression is complex. C/EBPβ (LAP1) can promote mammary epithelial cell differentiation [36], whereas C/EBPβ (LAP2) transforms normal mammary epithelial cells and induces EMT in culture [24]; LIP also promotes EMT in breast cancer and esophageal squamous cell carcinomas [18, 21, 24]. Dysregulation of C/EBPβ is markedly correlated with the malignancy of several tumors, including gliomas, Wilm's tumors and renal cell carcinomas [37-39]. In the present study, we analyzed the protein expression of LAP1, LAP2 and LIP in human primary HCC samples and showed that although LAP1 and LAP2 could be detected, the expression of LIP was barely detectable using the same conditions. Moreover, the LAP1 protein was significantly downregulated in HCC tissues compared with matched non-cancerous liver tissues, whereas LAP2 protein levels were similar. These findings indicate that LAP1 plays an important role in the regulation of ORM2 and HCC progression.

C/EBPβ upregulates ORM2 expression by directly bind to its promoter region [15]. The three isoforms of C/EBPβ have different transcriptional activity [23]. In this study, we verified that both LAP1 and LAP2 could activate the ORM2 promoter and upregulate ORM2 expression, while LIP had no effect on ORM2 promoter activity. Subsequent experiments showed that LAP1 repressed the *in vitro* migration and invasion of HCC cells via regulation of ORM2 expression. Moreover, C/EBPβ expression positively correlated with ORM2 expression in HCC tissues.

In conclusion, our findings demonstrate that ORM2 represses the metastatic potential of HCC cells *in vivo* and *in vitro*. LAP1/2 activates ORM2 expression via directly binding to the ORM2 promoter and repressing the *in vitro* migration and invasion of HCC cells at least partially through ORM2. Thus, these results provide novel potential targets for the treatment and prevention of HCC metastasis.

## MATERIALS AND METHODS

### Cell lines and cell culture

The PLC/PRF/5, Hep3B, and SK-Hep-1 HCC cell lines were obtained from the American Type Culture Collection (Manassas, VA, USA). The Huh7 cell line was purchased from the Riken Cell Bank (Tsukuba, Japan). The SMMC-7721 cell line was purchased from the cell bank of the Institute of Biochemistry and Cell Biology of the Chinese Academy of Sciences (Shanghai, China). The MHCC-97L, MHCC-97H and MHCC-LM3 cell lines were kindly provided by the Liver Cancer Institute, Zhongshan Hospital of Fudan University (Shanghai, China). These cell lines were cultured in Dulbecco's modified Eagle's medium (DMEM; Sigma-Aldrich, St. Louis, MO, USA) with 10% fetal bovine serum (FBS; Hyclone, Logan, UT, USA) at 37°C in 5% CO_2_. The HCC-LY5 cell line was established in our laboratory.

### Quantitative real-time polymerase chain reaction (qRT-PCR)

Total RNA was extracted using TRIzol reagent (Invitrogen, Carlsbad, CA, USA). Reverse transcription was performed using the PrimeScript^TM^ RT Reagent Kit (TaKaRa, Dalian, China). The qRT-PCR primers are provided in Supplementary Table 1.

### Western blot

Proteins extracted from cell lysates and tissue lysates were separated on 10% SDS-PAGE and transferred onto nitrocellulose membranes according to the manufacturer's instructions (Sigma-Aldrich, St. Louis, MO, USA). The anti-ORM2 polyclonal antibody (mab3694) was purchased from R&D Systems (Abingdon, UK), the anti-C/EBPβ polyclonal antibody (sc-150) was purchased from Santa Cruz Biotechnology (Santa Cruz, CA, USA) and the β-actin antibody (A3854) was purchased from Sigma-Aldrich (Sigma-Aldrich, St. Louis, MO, USA).

### Colony formation assays

For colony formation assays, 3000 cells per well were plated on 6-well plates and cultured for 2 weeks, then fixed with 10% formaldehyde for 30 min at 37°C. The cells were stained with Giemsa solution. Finally, the cell colonies were quantified.

### MTT assays

For MTT assays, 3000 cells per well were plated on 96-well plates and incubated for 24 h. Then, 100 μL of MTT reagent (5 mg /ml, Sigma-Aldrich) was added to each well and incubated for 4 h at 37°C. The optical density (OD) value was recorded at a dual wavelength (570 nm, 630 nm) every day for 7 days.

### RNA interference-based knock down assays

Small-interfering RNA (siRNA) oligos targeting ORM2 and a negative control (Cat. No. B01001) were synthesized by GenePharma (Shanghai, China). Three fragments were designed to target the corresponding gene transcripts, and the silencing effects of the sequences were verified by qRT-PCR. The sequences of the siRNA targeting ORM2 are as follows: siORM2-1: 5′-GAAACGAGGAGUACAAUAATT-3′; siORM2-2: 5′-GCUUCUAUAACUCCAGUUATT-3′; and siORM2-3: 5′-CCAGGUCAGAUGUCAUGUATT-3′.

### *In vitro* migration and invasion assays

A total of 1×10^6^ cells were seeded into the upper chamber of a transwell (BD Biosciences, NJ, USA) in serum-free media, while the lower chamber of the transwell contained DMEM with 10% FBS. After 12 h or 36 h of incubation, the cells in the upper chamber were removed. The cells were fixed with 10% formaldehyde for 30 min, stained using Giemsa solution and quantified.

### *In vivo* metastasis assays

Six-week-old BALB/C-nu/nu nude male mice were randomly divided into groups. All animals were maintained under specific pathogen-free conditions. For the *in vivo* tumor metastasis assay, 1×10^6^ cells stably expressing ORM2 and the pWPXL-control were orthotopically injected into the left hepatic lobe. After 4 weeks, all mice were euthanized. The livers and lungs were collected and fixed in 10% neutral phosphate-buffered formalin. The samples were embedded in paraffin and stained with hematoxylin and eosin. The experiments were performed according to the guidelines approved by the Shanghai Medical Experimental Animal Care Commission.

### Plasmid constructs, lentivirus production, and cell transfection

Full-length human ORM2, LAP1, LAP2 and LIP gene sequences were PCR amplified and cloned into pWPXL (Addgene, Cambridge, MA, USA) at the *Bam*HI and *Eco*RI sites. The ORM2 full-length promoter sequence was amplified from −1500 bp to +150 bp; the deleted sequence represented the region from −500 bp and −300 bp to +150 bp. The mutant was generated to delete the DNA binding site (−48 to −32) [15]. All promoter sequences were cloned into the pGL3-enhancer vector (Promega, Madison, WI, USA) at the *Kpn*I and *Hin*dIII sites. The primers used for cloning and testing are provided in Supplementary Table 2.

### Luciferase reporter assay

293T cells were plated in 96-well culture plates for 24 h and transfected with the relevant constructs. *Renilla* and firefly luciferase activity was determined according to the manufacturer's instructions (Promega).

### Chromatin immunoprecipitation (ChIP)

The ChIP assay was performed in SMMC-7721-pWPXL, SMMC-7721-C/EBPβ, Li-7-pWPXL, and Li-7- C/EBPβ cells. The cells were cross-linked with 10% formaldehyde for 10 min at 37°C, and then reversed with 1 M glycine for 5 min at 37°C. Then, after washing with 1× PBS buffer, the cells were harvested in Tissue Protein Extraction Reagent (Thermo Scientific), incubated on ice for 5 min, and centrifuged at 2, 000 × *g* for 5 min. The sediments were suspended in nuclei lyses buffer, and the DNA was crushed into fragments of 1, 000 base pairs by sonication. Antibodies against C/EBPβ (Santa Cruz Biotechnology, Inc. USA) were added using protein A/G agarose beads (Sigma-Aldrich) and incubated overnight at 4°C. After reversing the crosslink, the DNA was isolated and used for polymerase chain reaction (PCR) analysis. Primers for the PCR of the ORM2 promoter are as follows: forward, 5′-AAATCTGTGGACTCACACG-3′ and reverse, 5′-TGACACAATCCTGCCAG-3′.

### Immunohistochemistry

The 236 pairs of HCC tissue samples were obtained from the Qidong Liver Cancer and stored at −80°C. Anti-ORM2 polyclonal antibody (ab16046) was purchased from Abcam (Cambridge, UK), and the anti-C/EBPβ polyclonal antibody (sc-150) was purchased from Santa Cruz Biotechnology, Inc. The immunohistochemistry and signal evaluation were performed according to our previously described procedures [40]. Informed consent was obtained from all patients, and the study was approved by the Ethics Committee of Shanghai Jiao Tong University.

### Statistical analysis

Statistical analyses were performed using SPSS (Statistical Package for the Social Sciences) 13.0 software. The results are presented as the mean±SD and were compared using Student's *t*-test. The χ^2^ test was used for categorical data. Statistical computations were performed using GraphPad Prism version 5.0. *P* < 0.05 was considered significant. *P < 0.05; **P < 0.01, ***P < 0.001.

## SUPPLEMENTARY MATERIAL TABLES AND FIGURES



## References

[1] Marquardt JU, Thorgeirsson SS (2014). SnapShot: Hepatocellular carcinoma. Cancer Cell.

[2] Padua D, Zhang XH, Wang Q, Nadal C, Gerald WL, Gomis RR, Massague J (2008). TGFbeta primes breast tumors for lung metastasis seeding through angiopoietin-like 4. Cell.

[3] Hochepied T, Berger FG, Baumann H, Libert C (2003). Alpha(1)-acid glycoprotein: an acute phase protein with inflammatory and immunomodulating properties. Cytokine Growth Factor Rev.

[4] Mestriner FL, Spiller F, Laure HJ, Souto FO, Tavares-Murta BM, Rosa JC, Basile-Filho A, Ferreira SH, Greene LJ, Cunha FQ (2007). Acute-phase protein alpha-1-acid glycoprotein mediates neutrophil migration failure in sepsis by a nitric oxide-dependent mechanism. Proc Natl Acad Sci U S A.

[5] Pos O, Oostendorp RA, van der Stelt ME, Scheper RJ, Van Dijk W (1990). Con A-nonreactive human alpha 1-acid glycoprotein (AGP) is more effective in modulation of lymphocyte proliferation than Con A-reactive AGP serum variants. Inflammation.

[6] Ligresti G, Aplin AC, Dunn BE, Morishita A, Nicosia RF (2012). The acute phase reactant orosomucoid-1 is a bimodal regulator of angiogenesis with time- and context-dependent inhibitory and stimulatory properties. PLoS One.

[7] Muchitsch EM, Teschner W, Linnau Y, Pichler L (1996). *In vivo* effect of alpha 1-acid glycoprotein on experimentally enhanced capillary permeability in guinea-pig skin. Arch Int Pharmacodyn Ther.

[8] Miranda-Ribera A, Passaniti A, Ceciliani F, Goldblum SE (2014). alpha1-acid glycoprotein disrupts capillary-like tube formation of human lung microvascular endothelia. Exp Lung Res.

[9] Cai B, Fan J, Zeng M, Zhang L, Fu BM (2012). Adhesion of malignant mammary tumor cells MDA-MB-231 to microvessel wall increases microvascular permeability via degradation of endothelial surface glycocalyx. J Appl Physiol (1985).

[10] Zhang X, Xiao Z, Liu X, Du L, Wang L, Wang S, Zheng N, Zheng G, Li W, Dong Z, Zhuang X, Wang C (2012). The potential role of ORM2 in the development of colorectal cancer. PLoS One.

[11] Gao F, Zhang X, Whang S, Zheng C (2014). Prognostic Impact of Plasma ORM2 Levels in Patients with Stage II Colorectal Cancer. Ann Clin Lab Sci.

[12] Asao T, Yazawa S, Nishimura T, Hayashi T, Shimaoka H, Saniabadi AR, Kuwano H (2013). Development of a novel system for mass spectrometric analysis of cancer-associated fucosylation in plasma alpha1-acid glycoprotein. Biomed Res Int.

[13] Ferens-Sieczkowska M, Kratz EM, Kossowska B, Passowicz-Muszynska E, Jankowska R (2013). Comparison of haptoglobin and alpha(1)-acid glycoprotein glycosylation in the sera of small cell and non-small cell lung cancer patients. Postepy Hig Med Dosw (Online).

[14] Rucksaken R, Khoontawad J, Roytrakul S, Pinlaor P, Hiraku Y, Wongkham C, Pairojkul C, Boonmars T, Pinlaor S (2012). Proteomic analysis to identify plasma orosomucoid 2 and kinesin 18A as potential biomarkers of cholangiocarcinoma. Cancer Biomark.

[15] Sai K, Kurose K, Koizumi T, Katori N, Sawada J, Matsumura Y, Saijo N, Yamamoto N, Tamura T, Okuda H, Saito Y (2014). Distal promoter regions are responsible for differential regulation of human orosomucoid-1 and -2 gene expression and acute phase responses. Biol Pharm Bull.

[16] Timchenko LT, Salisbury E, Wang GL, Nguyen H, Albrecht JH, Hershey JW, Timchenko NA (2006). Age-specific CUGBP1-eIF2 complex increases translation of CCAAT/enhancer-binding protein beta in old liver. J Biol Chem.

[17] Zahnow CA, Younes P, Laucirica R, Rosen JM (1997). Overexpression of C/EBPbeta-LIP, a naturally occurring, dominant-negative transcription factor, in human breast cancer. J Natl Cancer Inst.

[18] Park BH, Kook S, Lee S, Jeong JH, Brufsky A, Lee BC (2013). An isoform of C/EBPbeta, LIP, regulates expression of the chemokine receptor CXCR4 and modulates breast cancer cell migration. J Biol Chem.

[19] Johansson J, Berg T, Kurzejamska E, Pang MF, Tabor V, Jansson M, Roswall P, Pietras K, Sund M, Religa P, Fuxe J (2013). MiR-155-mediated loss of C/EBPbeta shifts the TGF-beta response from growth inhibition to epithelial-mesenchymal transition, invasion and metastasis in breast cancer. Oncogene.

[20] Lu WC, Kao SY, Yang CC, Tu HF, Wu CH, Chang KW, Lin SC (2014). EGF up-regulates miR-31 through the C/EBPbeta signal cascade in oral carcinoma. PLoS One.

[21] Li J, Shan F, Xiong G, Chen X, Guan X, Wang JM, Wang WL, Xu X, Bai Y (2014). EGF-induced C/EBPbeta participates in EMT by decreasing the expression of miR-203 in esophageal squamous cell carcinoma cells. J Cell Sci.

[22] Choudhury M, Qadri I, Rahman SM, Schroeder-Gloeckler J, Janssen RC, Friedman JE (2011). C/EBPbeta is AMP kinase sensitive and up-regulates PEPCK in response to ER stress in hepatoma cells. Mol Cell Endocrinol.

[23] Descombes P, Schibler U (1991). A liver-enriched transcriptional activator protein, LAP, and a transcriptional inhibitory protein, LIP, are translated from the same mRNA. Cell.

[24] Bundy LM, Sealy L (2003). CCAAT/enhancer binding protein beta (C/EBPbeta)-2 transforms normal mammary epithelial cells and induces epithelial to mesenchymal transition in culture. Oncogene.

[25] Lee YJ, Huang X, Kropat J, Henras A, Merchant SS, Dickson RC, Chanfreau GF (2012). Sphingolipid signaling mediates iron toxicity. Cell Metab.

[26] Katori N, Sai K, Saito Y, Fukushima-Uesaka H, Kurose K, Yomota C, Kawanishi T, Nishimaki-Mogami T, Naito M, Sawada J, Kunitoh H, Nokihara H, Sekine I (2011). Genetic variations of orosomucoid genes associated with serum alpha-1-acid glycoprotein level and the pharmacokinetics of paclitaxel in Japanese cancer patients. J Pharm Sci.

[27] Lee YS, Choi JW, Hwang I, Lee JW, Lee JH, Kim AY, Huh JY, Koh YJ, Koh GY, Son HJ, Masuzaki H, Hotta K, Alfadda AA (2010). Adipocytokine orosomucoid integrates inflammatory and metabolic signals to preserve energy homeostasis by resolving immoderate inflammation. J Biol Chem.

[28] Spiller F, Carlos D, Souto FO, de Freitas A, Soares FS, Vieira SM, Paula FJ, Alves-Filho JC, Cunha FQ (2012). alpha1-Acid glycoprotein decreases neutrophil migration and increases susceptibility to sepsis in diabetic mice. Diabetes.

[29] Grivennikov SI, Greten FR, Karin M (2010). Immunity, inflammation, and cancer. Cell.

[30] Sorensson J, Matejka GL, Ohlson M, Haraldsson B (1999). Human endothelial cells produce orosomucoid, an important component of the capillary barrier. Am J Physiol.

[31] Steeg PS (2006). Tumor metastasis: mechanistic insights and clinical challenges. Nat Med.

[32] Irmak S, Oliveira-Ferrer L, Singer BB, Ergun S, Tilki D (2009). Pro-angiogenic properties of orosomucoid (ORM). Exp Cell Res.

[33] Fan C, Nylander PO, Stendahl U, Thunell M, Beckman L (1995). Synergistic interaction between ORM1 and C3 types in disease associations. Exp Clin Immunogenet.

[34] Alam T, An MR, Mifflin RC, Hsieh CC, Ge X, Papaconstantinou J (1993). trans-activation of the alpha 1-acid glycoprotein gene acute phase responsive element by multiple isoforms of C/EBP and glucocorticoid receptor. J Biol Chem.

[35] An MR, Hsieh CC, Reisner PD, Rabek JP, Scott SG, Kuninger DT, Papaconstantinou J (1996). Evidence for posttranscriptional regulation of C/EBPalpha and C/EBPbeta isoform expression during the lipopolysaccharide-mediated acute-phase response. Mol Cell Biol.

[36] Liu Q, Boudot A, Ni J, Hennessey T, Beauparlant SL, Rajabi HN, Zahnow C, Ewen ME (2014). Cyclin D1 and C/EBPbeta LAP1 operate in a common pathway to promote mammary epithelial cell differentiation. Mol Cell Biol.

[37] Li W, Kessler P, Yeger H, Alami J, Reeve AE, Heathcott R, Skeen J, Williams BR (2005). A gene expression signature for relapse of primary wilms tumors. Cancer Res.

[38] Oya M, Horiguchi A, Mizuno R, Marumo K, Murai M (2003). Increased activation of CCAAT/enhancer binding protein-beta correlates with the invasiveness of renal cell carcinoma. Clin Cancer Res.

[39] Homma J, Yamanaka R, Yajima N, Tsuchiya N, Genkai N, Sano M, Tanaka R (2006). Increased expression of CCAAT/enhancer binding protein beta correlates with prognosis in glioma patients. Oncol Rep.

[40] Li H, Ge C, Zhao F, Yan M, Hu C, Jia D, Tian H, Zhu M, Chen T, Jiang G, Xie H, Cui Y, Gu J (2011). Hypoxia-inducible factor 1 alpha-activated angiopoietin-like protein 4 contributes to tumor metastasis via vascular cell adhesion molecule-1/integrin beta1 signaling in human hepatocellular carcinoma. Hepatology.

