# Supplementary material for: Orosomucoid 2 inhibits tumor metastasis and is upregulated by CCAAT/enhancer binding protein β in hepatocellular carcinomas
Source: Oncotarget. 2015 Apr 19;6(18):16106–19. doi: 10.18632/oncotarget.3867 (PMC4599259; doi:10.18632/oncotarget.3867)
Supplement: Supplementary file 1 [file oncotarget-06-16106-s001.pdf]

Orosomucoid 2 inhibits tumor metastasis and is upregulated by CCAAT/enhancer binding protein β in hepatocellular carcinomas

Supplementary Material

Supplementary Table 1. The PCR primers for qRT-PCR.

ORM2	Forward	TCGGTTCAGGAGATCCAAGC
	Reverse	GAACAGCAGG GAGCAACAT
C/EBPβ	Forward	TCCAAACCAACCGCACAT
	Reverse	GCAGAGGGAGAAGCAGAGAG

Supplementary Table 2. Primers for vector constructs.

ORM2	Forward	CGCGGATCCATGGCGCTGTCCTGGGTTC
	Reverse	CCGGAATTCCTAGGATTCCCCCTCCTCCT
ORM2-promoter -1500bp	Forward	CGCGGTACCGTAGAGGAAGCAAAAAAC
	Reverse	CCGAAGCTTCTGTAAGAACCCAGGACA
ORM2-promoter -500bp	Forward	CGCGGTACCATTGGTGAAAGAGAAGCAA
	Reverse	CCGAAGCTTCTGTAAGAACCCAGGACA
ORM2-promoter -300bp	Forward	CGCGGTACCCCACAACATACATCCCAT
	Reverse	CCGAAGCTTCTGTAAGAACCCAGGACA
ORM2-promoter -mutant	Big primers	GTTTGGAGGCGGTGCTCCGAGCTGGCCTTTCATTTCCC AAGTGCTGGCAGGATTGTGTCA
C/EBPβ (LAP1)	Forward	CGCGGATCCATGCAACGCCTGGTGGCCT
	Reverse	CCGGAATTCCTAGCAGTGGCCGGAGGAG
C/EBPβ (LAP2)	Forward	CGCGGATCCATGGAAGTGGCCAACTTCT
	Reverse	CCGGAATTCCTAGCAGTGGCCGGAGGAG
C/EBPβ (LIP)	Forward	CGCGGATCCATGGCGGCGGGCTTCCCGT
	Reverse	CCGGAATTCCTAGCAGTGGCCGGAGGAG
sh-ORM2-1	Forward	CGCGTCCCCGAAACGAGGAGTACAATAATTCAAGAGAT TATTGTACTCCTCGTTTCTTTTTGGAAAT
	Reverse	CGATTTCCAAAAAGAAACGAGGAGTACAATAATCTCTTG AATTATTGTACTCCTCGTTTCGGGGA
sh-ORM2-2	Forward	CGCGTCCCCGCTTCTATAACTCCAGTTATTCAAGAGATAA CTGGAGTTATAGAAGCTTTTTGGAAAT
	Reverse	CGATTTCCAAAAAGCTTCTATAACTCCAGTTATCTCTTGA ATAACTGGAGTTATAGAAGCGGGGA

A

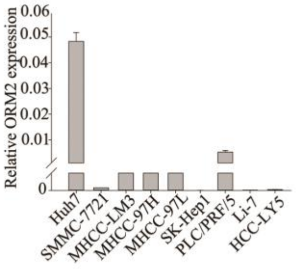

Supplementary Figure 1

The expression of ORM2 in HCC cell lines. (a) qRT-PCR was performed to detect ORM2 expression in HCC cell lines.

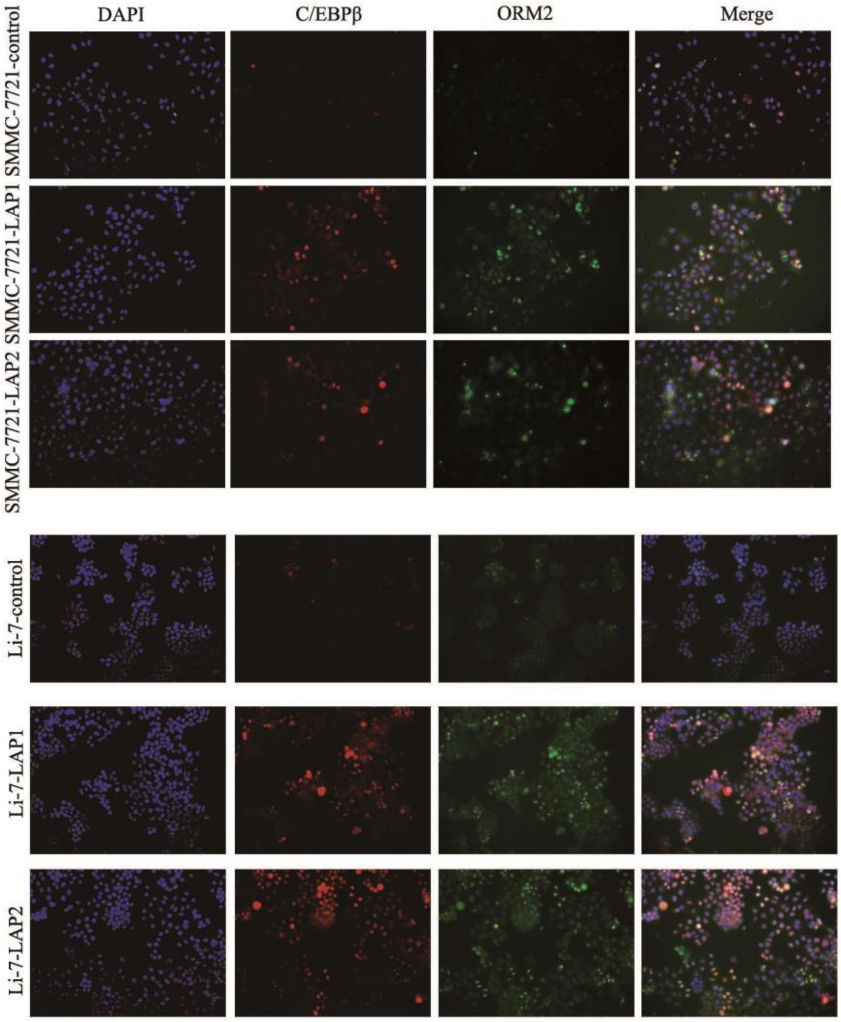

Supplementary Figure 2

Immunofluorescent co-staining of LAP1/2 and ORM2 proteins in HCC cell lines. Immunofluorescence assays of co-expression of LAP1/2 (red) and ORM2 (green) proteins were carried out in SMMC-7721 and Li-7 cells with stably LAP1/2 overexpression (SMMC-7721-LAP1, -LAP2; Li-7-LAP1, -LAP2) and in the untreated control cells (SMMC-7721-control; Li-7- control). DAPI was used to counter-staining for nuclei (blue).

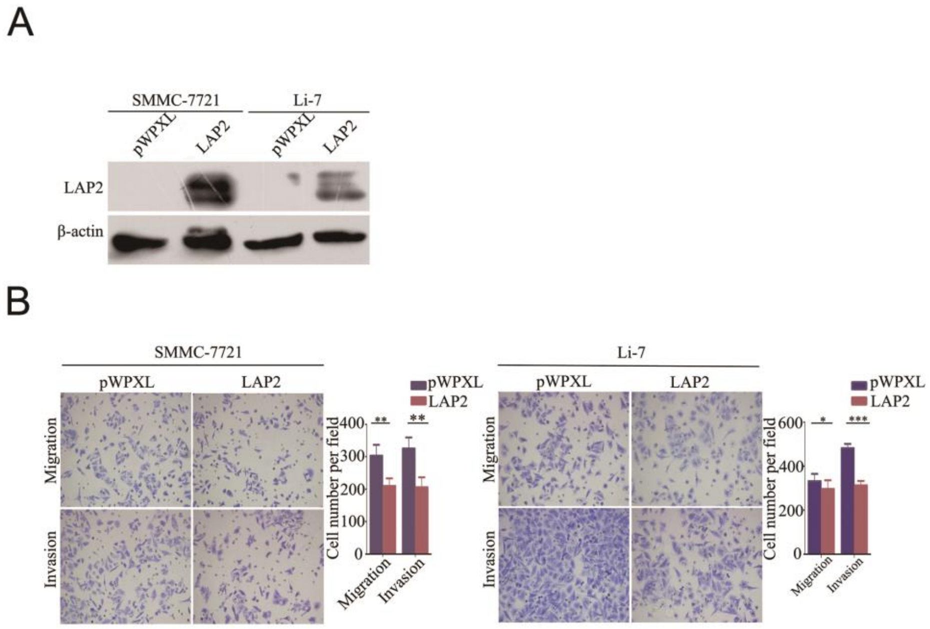

Supplementary Figure 3

LAP2 represses HCC cell migration and invasion ability *in vitro*. (a) Western blotting of LAP2 protein levels in SMMC-7721 and Li-7 cells stably transfected with LAP2 or the control. (b)The migration and invasion ability of SMMC-7721 and Li-7 cells transfected with LAP2 were assessed by transwell assays; cells transfected with the empty vector were used as a control. *, p<0.05, **, p<0.01, ***, p<0.001.
